# Integrated contra-directionally coupled chirped Bragg grating waveguide with a linear group delay spectrum

**DOI:** 10.1007/s12200-023-00061-8

**Published:** 2023-04-10

**Authors:** Xudong Gao, Zhenzhu Xu, Yupeng Zhu, Chengkun Yang, Shoubao Han, Zongming Duan, Fan Zhang, Jianji Dong

**Affiliations:** 1 Anhui Province Engineering Laboratory for Antennas and Microwave, East China Research Institute of Electronic Engineering, Hefei, 230000 China; 2 Wuhan National Laboratory for Optoelectronics, Huazhong University of Science and Technology, Wuhan, 430074 China

**Keywords:** Bragg gratings, Silicon photonics, True time delay

## Abstract

Due to the advantages of low propagation loss, wide operation bandwidth, continuous delay tuning, fast tuning speed, and compact footprints, chirped Bragg grating waveguide has great application potential in wideband phased array beamforming systems. However, the disadvantage of large group delay error hinders their practical applications. The nonlinear group delay spectrum is one of the main factors causing large group delay errors. To solve this problem, waveguides with nonlinear gradient widths are adopted in this study to compensate for the nonlinear effect of the grating apodization on the mode effective index. As a result, a linear group delay spectrum is obtained in the experiment, and the group delay error is halved.

**Graphical Abstract:**

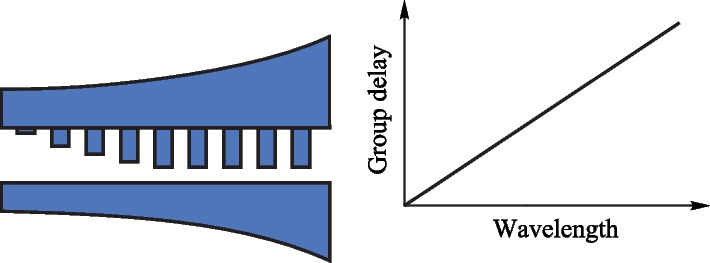

**Supplementary Information:**

The online version contains supplementary material available at 10.1007/s12200-023-00061-8.

## Introduction

Phased array technology has important applications in radar and electronic countermeasure systems. At present, phased array radar is developing toward the use of higher frequency and wider bandwidth, but the bottleneck restricting its broadband characteristics comes from wideband phased array beamforming systems. In the traditional phased array system based on phase shifters, beam dispersion occurs in the wideband operation mode owing to the correlation between phase and frequency. In contrast, phased array systems based on true time delay can achieve broadband beamforming because the time delay is frequency-independent [1]. True time delay can be achieved by means of electric delay or microwave photonic delay. The electric delay chip has been widely used in phased array beamforming systems [2]; however, in the case of ultra-wideband, the crosstalk is serious owing to the high return loss, resulting in a large group delay error (GDE). Microwave photonic delay can be realized by electro-optic modulation, optical time delay, and photoelectric demodulation [3, 4]. Because photonic devices generally exhibit low return loss, the GDE of microwave photonic delay is expected to be small. Furthermore, it is potential to realize a microwave photonic delay system on a chip through hybrid integration techniques, as the integrated high-bandwidth electro-optic modulators [5, 6] and detectors [7, 8] have already been demonstrated in recent years. Therefore, integrated optical true time delay lines have recently attracted extensive research interest.

Integrated optical true time delay lines can be implemented using various approaches such as switches, optical ring resonators (ORRs), photonic crystal waveguides (PhCWs), and chirped Bragg grating waveguides. In switch-based delay lines, discrete delays can be realized by connecting switches with waveguides of different lengths and the time delay can be tuned by changing the switching state [1, 9, 10]. This method can achieve accurate delay, but the difficulty lies in the calibration of switches. Recently, calibration-free Mach–Zehnder switches have been implemented by introducing novel tapered Euler S-bends with a wide core and incorporating bent asymmetric directional coupler mode filters, paving the way toward the real application of switch-based delay lines [11]. ORRs have the advantages of continuous delay tuning and compact footprints. However, the operation bandwidth is limited by the delay-bandwidth product of a single ORR. To obtain a large delay with wide operation bandwidth, cascaded ORRs have been proposed. However, the tuning of cascaded ORRs become more difficult as well [12, 13]. PhCWs have a compact footprint owing to their strong optical confinement and slow-light effect [14, 15]. One-dimensional fishbone photonic crystal waveguides have been experimentally demonstrated to have lower optical propagation loss, high dispersion, and continuous delay tunability [14]. However, their intrinsic nonlinear group-delay spectrum limits the scope of their applications.

Chirped Bragg grating waveguides exhibit low propagation loss, wide operation bandwidth, continuous delay tuning, and compact footprints [16, 17]. Their tuning speed depends on the tunable laser and can reach the MHz level. Furthermore, the number of delay channels can be reduced by wavelength division multiplexing [4, 18] and channel-shared structures [19]. In the past several years, spiral and contra-directionally coupled Bragg grating waveguides with positive and negative dispersions have been fabricated, and multi-channel time-delay arrays have been developed based on these structures [20–23]. However, chirped Bragg gratings generally suffer from large GDE, which hinders their practical applications. The GDE originates, on one hand, from the ripples in the group delay spectra, which can be well suppressed by apodization [17, 20–23] and, on the other hand, from the nonlinear relation between the group delay and wavelength, which is induced by the nonlinear gradient of the mode effective index along the waveguide.

In this study, the width of the waveguide is nonlinearly corrected to solve the problem of the nonlinear delay spectrum of the contra-directionally coupled chirped Bragg grating waveguide. First, the nonlinear effect of grating apodization on the mode effective index is analyzed. Subsequently, the width of the waveguide is designed to have a nonlinear gradient to compensate for this nonlinear effect. Finally, a linear group delay spectrum is successfully obtained in the experiment.

## Principle and design

**Fig. 1:**
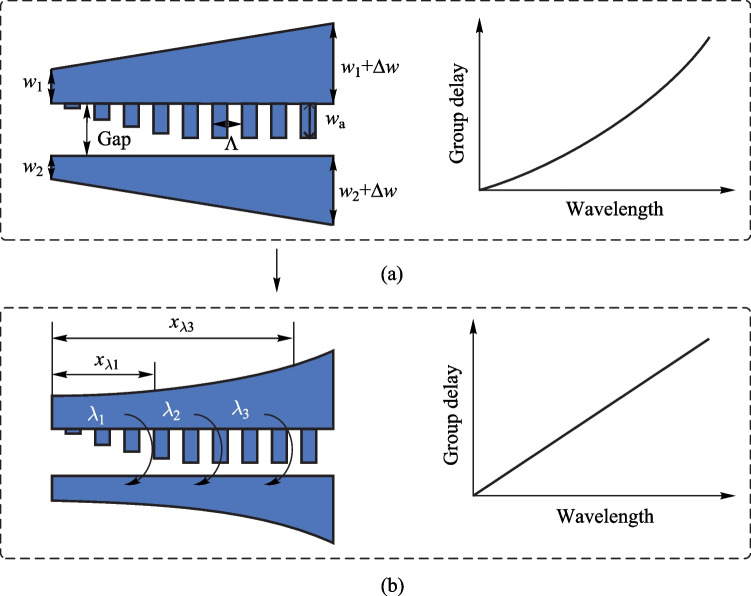
Schematic diagrams of the contra-directionally coupled chirped Bragg grating waveguide and group delay spectra. **a** Conventional structure has linearly graded waveguide width and shows a nonlinear group delay spectrum, **b** proposed structure has corrected graded waveguide width and shows a linear group delay spectrum

Schematics of the contra-directionally coupled chirped Bragg grating waveguide are shown in Fig. 1. Conventional structure, as shown in Fig. 1a, consists of two tapered strip waveguides with widths ranging from *w*_1_ and *w*_2_ to *w*_1_ + Δ*w* and *w*_2_ + Δ*w*, respectively. Bragg gratings with a period Λ, duty of 50%, and width *w*_a_ are introduced on the sidewall of the upper waveguide. To avoid bandwidth overlap between the contra-directionally coupling and back reflections, the width difference between the two waveguides must be large enough [23]. The main structure parameters in this study are set as follows: *w*_1_ = 584 nm, *w*_2_ = 484 nm, Δ*w* = 20 nm, gap = 200 nm, Λ = 300 nm, *w*_a_ = 50 nm, and the grating period number is 4800. To suppress the delay ripples, sinusoidal apodization of the gratings was applied over one-third of the entire grating length on the input side. The width of the apodized grating *w*_g_ can be expressed as follows:

1 $$w_{{\text{g}}} (x) = \left\{ {\begin{array}{*{20}c} {w_{{\text{a}}} \cdot \sin [x \cdot (3/L) \cdot ({\uppi }/2)],} \\ {w_{{\text{a}}} ,} \\ \end{array} } \right. \, \begin{array}{*{20}c} {x \le L/3,} \\ {x > L/3,} \\ \end{array}$$

where* L* is the whole length of the gratings; *x* is the location along the waveguide; *w*_a_ is the maximum grating width.

In the conventional design, the widths of tapered strip waveguides are set to increase linearly to achieve a linear increase in the mode effective index along the waveguide [21–23]. However, the grating apodization introduces a nonlinear change in the mode effective index, resulting in a nonlinear group delay spectrum. To obtain a linear group delay spectrum, we propose that the width of the tapered strip waveguide should be nonlinearly corrected to compensate for the effect of grating apodization on the mode effective index; the improved structure is shown in Fig. 1b. The design of the chirped Bragg grating waveguides with linear group delay is implemented by using the equations as follows:

2 $$\lambda_{{\text{c}}} \, = \, 2 \cdot n_{{{\text{eff}}}} \cdot \Lambda ,$$

3 $$n_{{{\text{eff}}}} = \left( {n_{1} + n_{2} } \right)/2,$$

4 $$n_{{{\text{eff}}}} = a \cdot x + n{}_{{{\text{eff}}_{{0}} }},$$

5 $$\lambda_{{\text{c}}} \, = \, 2 \cdot \Lambda \cdot a \cdot x + 2 \cdot \Lambda \cdot n_{{{\text{eff}}_{{0}} }} ,$$

6 $$l = 2 \cdot x = (\lambda_{{\text{c}}} - 2 \cdot \Lambda \cdot n_{{{\text{eff}}_{{0}} }} { )/(}\Lambda \cdot a),$$

7 $$t = l/v_{{\text{g}}} = (\lambda_{{\text{c}}} - 2 \cdot \Lambda \cdot n_{{{\text{eff}}_{{0}} }} { )/(}\Lambda \cdot a \cdot v_{{\text{g}}} ),$$

where *n*_1_, *n*_2_, and *n*_eff_ are the mode effective indexes of upper and lower waveguides and coupled chirped Bragg grating waveguide composed of the upper and lower waveguides; *λ*_c_ is the central wavelength reflected by the chirped Bragg gratings; *a* and $n_{{\text{eff}_{{0}}}}$ are constants; *x* is the location along the waveguide; *l* is the optical path length; *t* is the group delay; and *v*_g_ is the group velocity.

According to Eq. (2), *λ*_c_ changes with *n*_eff_ and Λ. For contra-directionally coupled chirped Bragg grating waveguides, *n*_eff_ is the average value of the mode effective indices of the upper and lower waveguides (Eq. (3)) [22, 23]^.^ In this study, Λ is set as a constant, and *n*_eff_ is designed to increase linearly with *x* (Eq. (4)). Thus, *λ*_c_ also increases linearly with *x* (Eq. (5)). In Eq. (4), positive dispersion is obtained when *a* is positive; conversely, negative dispersion occurs when *a* is negative. As shown in Fig. 1b, light of different wavelengths is reflected at different positions in the waveguide, and the reflection position *x*_*λ*_ is proportional to the wavelength *λ*, according to Eq. (5). For the Bragg gratings, the optical path length* l* is twice of *x*_*λ*_ (Eq. (6)). Thus, the group delay can be calculated using Eq. (7). Supplementary Information: Fig. S1 shows the simulation results of *v*_g_ at the two ends of the contra-directionally coupled chirped Bragg grating waveguide. *v*_g_ at the two ends are approximately 7.13 at 1553 nm and 7.18 at 1562 nm with a variation of only ± 0.35%; therefore, *v*_g_ can be considered as a constant parameter. According to Eq. (7), the group delay, *t*, also increases linearly with the wavelength.

**Fig. 2:**
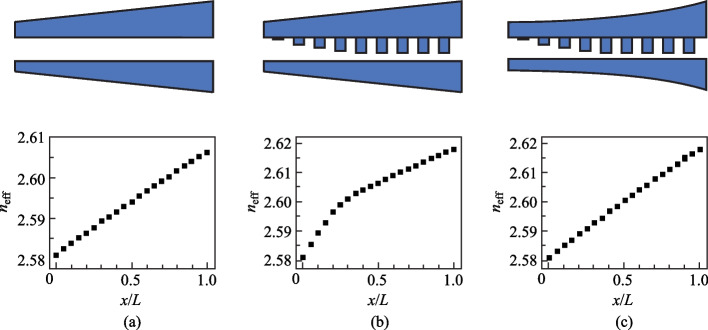
Simulated mode effective index of the waveguides. **a** The waveguide width increases linearly from 484 to 504 nm for the upper one and 584–604 nm for the lower one; **b** the waveguides have linearly increased width with apodized grating, grating apodization follows Eq. (1) and the maximum *w*_a_ is 50 nm; **c** the waveguides have nonlinearly corrected width with apodized gratings

In the simulation of strip waveguides, it is found that the mode effective index does not linearly depend on the waveguide width in the large sweeping range of the waveguide width (Supplementary Information: Fig. S2a). However, the relationship between them tends to be linear within a small width range of 20 nm, as shown in Supplementary Information: Fig. S2b and S2c. Thus, the mode effective index of the coupled waveguide calculated using Eq. (3) is also almost linear with increasing width. For waveguides with linearly increasing widths, as shown in Fig. 2a, *n*_eff_ also increases almost linearly with the location along the waveguide (Fig. 2a). However, it tends to be nonlinear when apodized gratings are added to the upper waveguide (Fig. 2b). Thus, it is confirmed that the apodized grating can induce a nonlinear change of *n*_eff_ along the waveguide. For the mode effective index simulation in Fig. 2b, the effective widths of the two tapered waveguides, *w*_up_eff_ and *w*_down_eff_ are expressed as follows:

8 $$w_{{{\text{up\_eff}}}} (x) = w_{1} + \beta \cdot w_{{\text{g}}} (x)/4 + \Delta w \cdot x/L,$$

9 $$w_{{{\text{down\_eff}}}} (x) = w_{2} + \beta \cdot w_{{\text{g}}} (x)/4 + \Delta w \cdot x/L,$$

where the constant parameter *β* is set to 0.8. To obtain a linearly increasing *n*_eff_, a linear increase in the effective widths of the two tapered waveguides is required. Thus, the real widths of the two tapered waveguides *w*_up_ and *w*_down_ should be nonlinearly corrected as follows:

10 $$w_{{{\text{up}}}} (x) = w_{1} + (\beta \cdot w_{{\text{a}}} /4 + \Delta w) \cdot x/L - \beta \cdot w_{{\text{g}}} (x)/4,$$

11 $$w_{{{\text{down}}}} (x) = w_{2} + (\beta \cdot w_{{\text{a}}} /4 + \Delta w) \cdot x/L - \beta \cdot w_{{\text{g}}} (x)/4.$$

After nonlinear correction of the waveguide width, the n_eff_ curve tends to be linear, as shown in Fig. 2c, indicating a successful design.

The proposed gratings waveguides are fabricated on a commercial silicon-on-insulator (SOI) wafer with a 250 nm silicon layer and a 3 μm buried oxide layer. The waveguides on the top silicon layer are fabricated by electron beam lithography and silicon dry etching. A silicon dioxide layer is then deposited on the waveguide for encapsulation.

## Results and discussion

**Fig. 3:**
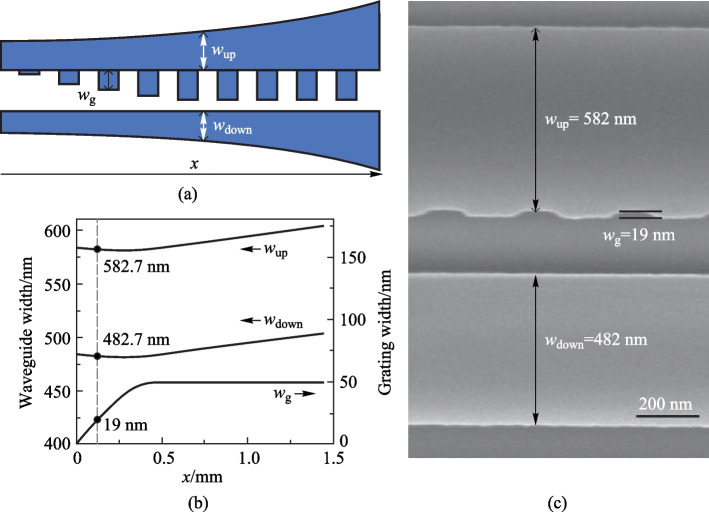
Chirped Bragg gratings waveguide after width nonlinear correction: **a** schematic diagram, **b** variation of the simulated *w*_up_, *w*_down_, and *w*_g_, and **c** SEM image

The waveguides after nonlinear width correction are shown in Fig. 3a. According to Eq. (1), the width of the apodized gratings *w*_g_ increases sinusoidally, which induces a rapid increase in the effective widths of the two tapered waveguides *w*_up_eff_ and *w*_down_eff_, according to Eqs. (8) and (9), resulting in an excessively rapid increase in *n*_eff_ (Fig. 2b). To compensate for this excessive increase in *w*_up_eff_ and *w*_down_eff_, the real widths of the two tapered waveguides, *w*_up_ and *w*_down_, need to be decreased slightly. As shown in Fig. 3b, when the grating width is 19 nm, *w*_up_ and *w*_down_ are 582.7 and 482.7 nm, respectively, which are smaller than the initial values of 584 and 484 nm, respectively. Figure 3c shows the SEM image of the waveguides in the apodization region. *w*_g_, *w*_up_, and *w*_down_ are 19, 582, and 482 nm, respectively and these values are consistent with those of the designed waveguide, as shown in Fig. 3b.

**Fig. 4:**
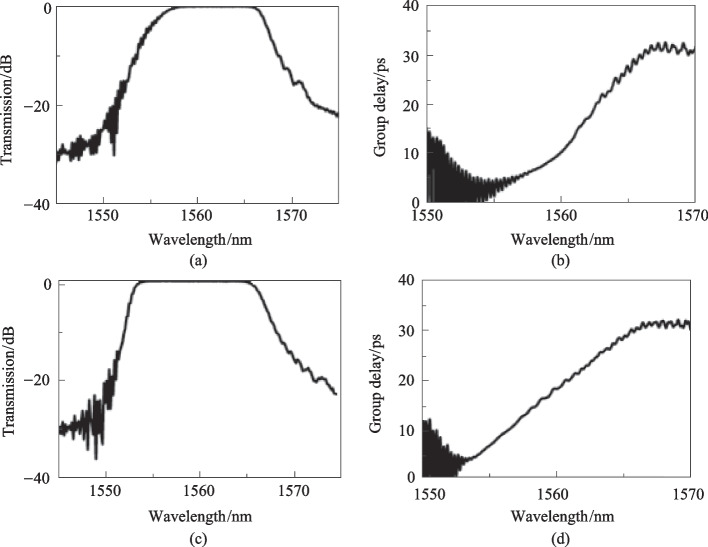
Simulated transmission and group delay spectra of the contra-directionally coupled chirped Bragg gratings waveguides with linearly increasing waveguide width (**a** and **b**), and nonlinearly corrected waveguide width (**c** and **d**)

Figure 4a and b show the simulated transmission and group delay spectra of the grating waveguide with a linearly increasing width of the strip waveguide, and the corresponding waveguide structures and simulated *n*_eff_ are shown in Fig. 2b. The rapidly increasing *n*_eff_ at the narrow waveguide side shown in Fig. 2b leads to a shorter coupling length per wavelength space, which in turn results in a low transmission at the short wavelength side of the transmission spectrum in Fig. 4a, as well as a slowly increasing group delay at the short wavelength side of the group delay spectrum in Fig. 4b. When the widths of the strip waveguide are nonlinearly corrected, as shown in Fig. 2c, the transmission at the short wavelength side increases (Fig. 4c) compared to that shown in Fig. 4a, and the group delay curve becomes linear across the transmission spectrum (Fig. 4d).

**Fig. 5:**
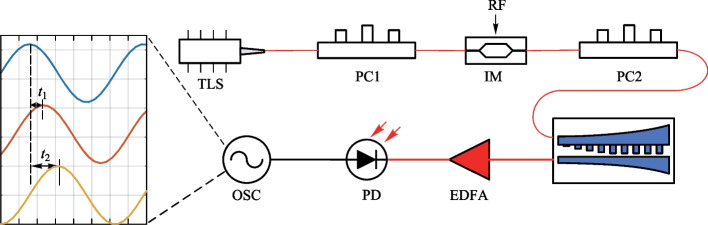
Measurement setup for group delay. *TLS* tunable laser source, *PC* polarization controller, *RF* radio frequency source, *IM* intensity modulator, *EDFA* erbium doped fiber amplifier, *PD* photodetector, *OSC* oscilloscope

The measurement setup for group delay is schematically shown in Fig. 5. A tunable laser is used to generate a light carrier, then a 10 GHz sinusoidal radio frequency (RF) signal is loaded on the light carrier through an intensity modulator (IM). The modulated signal is then injected into the fabricated contra-directionally coupled Bragg grating waveguides. Finally, the output signal is detected by a photodetector (PD) and analyzed by an oscilloscope (OSC). Due to the polarization dependence of IM and on-chip gratings coupler, two polarization controllers (PCs) are placed before the IM and the chip respectively to maximize the coupling efficiency. When the input wavelength changes within the passband of the coupled Bragg grating waveguides, the detected waveforms will have different time delays. By obtaining the time delay at different wavelengths, the group delay lines can be calculated.

**Fig. 6:**
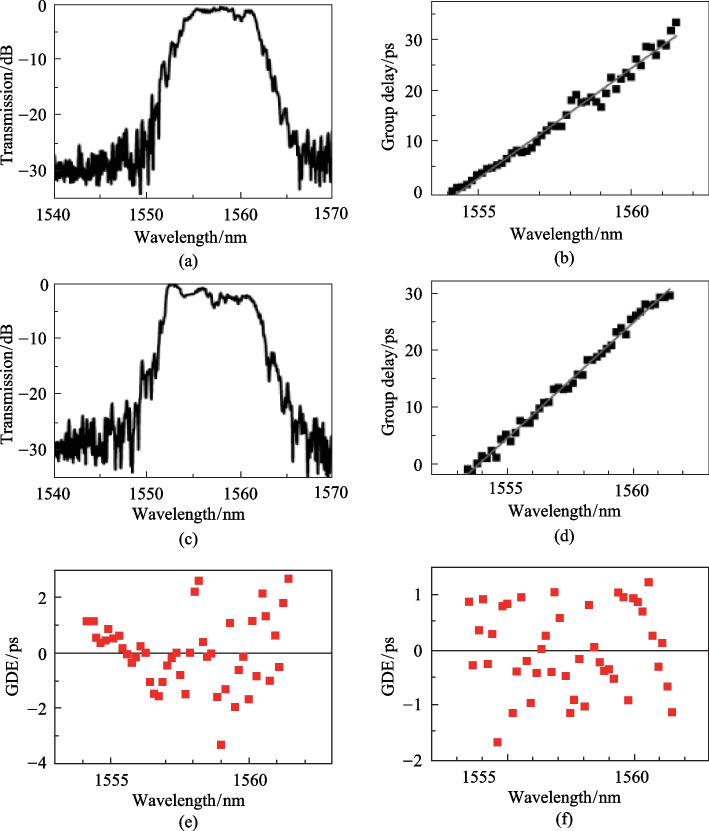
Measured transmission, group delay, and GDE spectra of the contra-directionally coupled chirped Bragg grating waveguides with linearly increasing waveguide width (**a**, **b** and **e**), and nonlinearly corrected waveguide width (**c**, **d** and **f**)

Figure 6 shows the measured transmission and group delay spectra of the grating waveguides with the structure shown in Fig. 2b and c. For both structures, the spectral bandwidth of the chirped Bragg grating waveguide is approximately 9 nm, the maximum group delay is approximately 30 ps, and the central wavelength is located at 1557.5 nm. The measurement results (Fig. 6a–d) agree well with the simulation results in Fig. 4a–d in terms of bandwidth and total group delay. The group delay line in Fig. 6b shows an upward bending trend, which is consistent with the simulated delay lines in Fig. 4b; this nonlinear bend produces a large GDE after linear fitting, where the GDE is calculated by the difference between the measured time delay and the linear fitted time delay. The measured average GDE is approximately ± 2 ps, which is approximately ± 7% of the total delay (Fig. 6e). In contrast, the group delay line in Fig. 6d is well matched linearly, and the measured average GDE is approximately ± 1 ps, which is approximately ± 4% of the total delay (Fig. 6f). Thus, it is proven that nonlinear correction of the waveguide width can effectively improve the linearity of the delay curve.

## Conclusions

This study is devoted to solving the problem of nonlinear delay spectrum of a contra-directionally coupled Bragg grating waveguide. Through the analysis of the mode effective index, it is found that grating apodization leads to a nonlinear gradient of the mode effective index along the waveguide, which then results in a nonlinear delay spectrum. To solve this problem, the width of the two strip waveguide in the coupled Bragg grating waveguides is nonlinearly corrected to compensate for the effect of the grating apodization on the mode effective index. As a result, a linear group delay spectrum is obtained in the experiment, and the GDE is halved compared the pre-correction case.

## Supplementary Information

Below is the link to the electronic supplementary material.

Supplementary file1 (PDF 87 KB)

## Data Availability

The data that support the findings of this study are available from the first author, upon reasonable request.

## References

[1] Yang H, Yun B (2020). A six bit silicon nitride optical true time delay line for Ka-band phased array antenna. J. Phys. Conf. Ser..

[2] Jeong J, Yom I, Kim J, Lee W, Lee CA (2018). 6–18-GHz GaAs multifunction chip with 8-bit true time delay and 7-bit amplitude control. IEEE Trans. Microw. Theory Tech..

[3] Choo G, Madsen CK, Palermo S, Entesari K (2018). Automatic monitor-based tuning of an RF silicon photonic 1×4 asymmetric binary tree true-time-delay beamforming network. J. Lightwave Technol..

[4] Ye X, Zhang F, Pan S (2016). Compact optical true time delay beamformer for a 2D phased array antenna using tunable dispersive elements. Opt. Lett..

[5] Wang C, Zhang M, Chen X, Bertrand M, Shams-Ansari A, Chandrasekhar S, Winzer P, Lončar M (2018). Integrated lithium niobate electro-optic modulators operating at CMOS-compatible voltages. Nature.

[6] He M, Xu M, Ren Y, Jian J, Ruan Z, Xu Y, Gao S, Sun S, Wen X, Zhou L, Liu L, Guo C, Chen H, Yu S, Liu L, Cai X (2019). High-performance hybrid silicon and lithium niobate Mach-Zehnder modulators for 100 Gbit s−1 and beyond. Nat. Photonics.

[7] Lischke S, Peczek A, Morgan JS, Sun K, Steckler D, Yamamoto Y, Korndörfer F, Mai C, Marschmeyer S, Fraschke M, Krüger A, Beling A, Zimmermann L (2022). Ultra-fast germanium photodiode with 3-dB bandwidth of 265 GHz. Nat. Photonics.

[8] Li M, Zhu N (2016). Recent advances in microwave photonics. Front. Optoelectron..

[9] Xie J, Zhou L, Li Z, Wang J, Chen J (2014). Seven-bit reconfigurable optical true time delay line based on silicon integration. Opt. Express.

[10] Wang X, Zhou L, Li R, Xie J, Lu L, Wu K, Chen J (2017). Continuously tunable ultra-thin silicon waveguide optical delay line. Optica.

[11] Song L, Chen T, Liu W, Liu H, Peng Y, Yu Z, Li H, Shi Y, Dai D (2022). Toward calibration-free Mach-Zehnder switches for next-generation silicon photonics. Photon. Res..

[12] Xiang C, Davenport ML, Khurgin JB, Morton PA, Bowers JE (2018). Low-loss continuously tunable optical true time delay based on Si3N4 ring resonators. IEEE J. Sel. Top. Quantum Electron..

[13] Lin D, Xu X, Zheng P, Yang H, Hu G, Yun B, Cui Y (2019). A tunable optical delay line based on cascaded silicon nitride microrings for Ka-band beamforming. IEEE Photonics J..

[14] Chung C, Xu X, Wang G, Pan Z, Chen RT (2018). On-chip optical true time delay lines featuring one-dimensional fishbone photonic crystal waveguide. Appl. Phys. Lett..

[15] Cassan E, Roux XL, Caer C, Hao R, Bernier D, Marris-Morini D, Vivien L (2011). Silicon slow light photonic crystals structures: present achievements and future trends. Front. Optoelectron. China.

[16] Kaushal S, Cheng R, Ma M, Mistry A, Burla M, Chrostowski L, Azaña J (2018). Optical signal processing based on silicon photonics waveguide Bragg gratings. Front Optoelectron..

[17] Du Z, Xiang C, Fu T, Chen M, Yang S, Bowers JE, Chen H (2020). Silicon nitride chirped spiral Bragg gratings with large group delay. APL Photonics.

[18] Ortega B, Mora J, Chulia R (2016). Optical beamformer for 2-D phased array antenna with subarray partitioning capability. IEEE Photonics J..

[19] Gao X, Zhu Y, Chong Y, Xu Z, Mei L, Cao J, Zhang F, Dong J (2021). Integrated channel-shared optical true time delay line array based on gratings-assisted contradirectional couplers for phased array antennas. Proc. SPIE.

[20] Sun Y, Wang D, Deng C, Lu M, Huang L, Hu G, Yun B, Zhang R, Li M, Dong J, Wang A, Cui Y (2021). Large group delay in silicon-on-insulator chirped spiral Bragg gratings waveguide. IEEE Photonics J..

[21] Shi W, Veerasubramanian V, Patel D, Plant DV (2014). Tunable nanophotonic delay lines using linearly chirped contradirectional couplers with uniform Bragg gratings. Opt. Lett..

[22] Wang X, Zhao Y, Ding Y, Xiao S, Dong J (2018). Tunable optical delay line based on integrated gratings-assisted contradirectional couplers. Photon. Res..

[23] Zhang F, Dong J, Zhu Y, Gao X, Zhang X (2020). Integrated optical true time delay network based on gratings-assisted contradirectional couplers for phased array antennas. IEEE J. Sel. Top. Quantum Electron..

