# Supplementary material for: Integrated contra-directionally coupled chirped Bragg grating waveguide with a linear group delay spectrum
Source: Front Optoelectron. 2023 Apr 10;16(1):6. doi: 10.1007/s12200-023-00061-8 (PMC10086080; doi:10.1007/s12200-023-00061-8)
Supplement: Supplementary file 1 — Supplementary file1 (PDF 87 KB) [file 12200_2023_61_MOESM1_ESM.pdf]

Supplementary Materials for:

Integrated contradirectional coupled chirped Bragg gratings waveguide with linear group delay spectrum

Xudong Gao[1], Zhenzhu Xu(✉)[1], Yupeng Zhu[1], Chengkun Yang[1], Shoubao Han[1], Zongming Duan[1], Fan Zhang[2], Jianji Dong[2]

1 Anhui Province Engineering Laboratory for Antennas and Microwave, East China Research Institute of Electronic Engineering, Hefei 230000, China

2 Wuhan National Laboratory for Optoelectronics, Huazhong University of Science and Technology, Wuhan 430074, China

✉zhenzhupearl@163.com

© The author(s) 2023

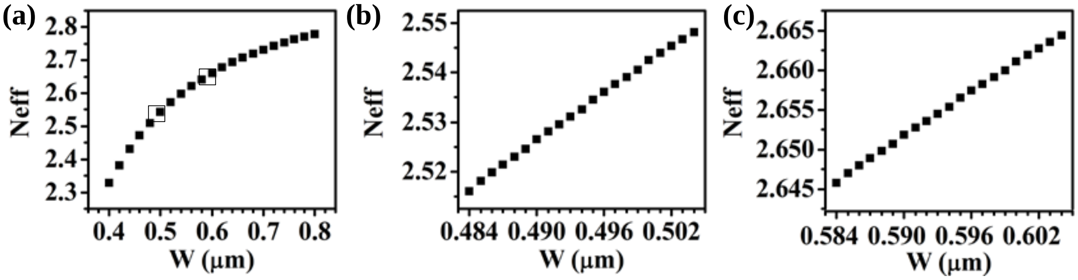

Fig. S1. Simulated mode effective index of the waveguide within a large range of waveguide width (a) and small range of waveguide width (b-c).

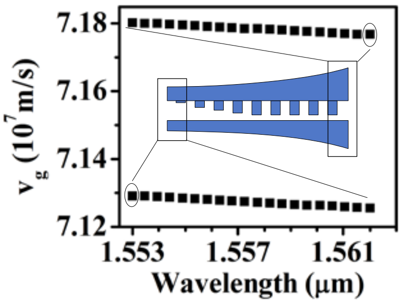

Fig. S2. Simulated group velocity of the contradirectional coupled chirped Bragg gratings waveguide.
